# Statistical learning of transition patterns in the songbird auditory forebrain

**DOI:** 10.1038/s41598-020-64671-4

**Published:** 2020-05-12

**Authors:** Mingwen Dong, David S. Vicario

**Affiliations:** Department of Psychology, Rutgers, the State University of New Jersey, New Brunswick, NJ United States

**Keywords:** Cortex, Sensory processing

## Abstract

Statistical learning of transition patterns between sounds—a striking capability of the auditory system—plays an essential role in animals’ survival (e.g., detect deviant sounds that signal danger). However, the neural mechanisms underlying this capability are still not fully understood. We recorded extracellular multi-unit and single-unit activity in the auditory forebrain of awake male zebra finches while presenting rare repetitions of a single sound in a long sequence of sounds (canary and zebra finch song syllables) patterned in either an alternating or random order at different inter-stimulus intervals (ISI). When preceding stimuli were regularly alternating (alternating condition), a repeated stimulus violated the preceding transition pattern and was a deviant. When preceding stimuli were in random order (control condition), a repeated stimulus did not violate any regularities and was not a deviant. At all ISIs tested (1 s, 3 s, or jittered at 0.8–1.2 s), deviant repetition enhanced neural responses in the alternating condition in a secondary auditory area (caudomedial nidopallium, NCM) but not in the primary auditory area (Field L2); in contrast, repetition suppressed responses in the control condition in both Field L2 and NCM. When stimuli were presented in the classical oddball paradigm at jittered ISI (0.8–1.2 s), neural responses in both NCM and Field L2 were stronger when a stimulus occurred as deviant with low probability than when the same stimulus occurred as standard with high probability. Together, these results demonstrate: (1) classical oddball effect exists even when ISI is jittered and the onset of a stimulus is not fully predictable; (2) neurons in NCM can learn transition patterns between sounds at multiple ISIs and detect violation of these transition patterns; (3) sensitivity to deviant sounds increases from Field L2 to NCM in the songbird auditory forebrain. Further studies using the current paradigms may help us understand the neural substrate of statistical learning and even speech comprehension.

## Introduction

In the natural environment, sounds often occur in complex temporal orders with variable intervening intervals (e.g., words in spoken speech). Transition patterns could characterize how likely one sound follows another sound in the sequences despite the variabilities in timing. Learning transition patterns is useful for predicting future stimuli, detecting deviant stimuli, and facilitating vocal communication^1–3^.

The auditory system can learn transition patterns between sounds without any external reinforcement. This phenomenon is called statistical learning and has been demonstrated in both humans and animals^3–5^. For example, after being exposed to sequences of tones with fixed transition patterns, human infants and adults showed surprise responses when they heard sequences that violated the transition patterns in the previously experienced sequences^4,6,7^. Similar statistical learning phenomena have also been reported in songbirds, monkeys and other animals^5,8,9^. However, most laboratory studies at the neural level used the oddball paradigm, in which one stimulus is presented with low probability as oddball while the other stimulus is presented with high probability as standard. Previous studies have shown that a stimulus elicits larger neural responses as an oddball than as a standard^10–13^. However, the enhanced neural responses to the oddball may be because the oddball is rare and therefore unexpected rather than because the oddball violates the repetition pattern of the standard. In addition, in the natural environment, the intervals between sounds are often variable instead of fixed and can span multiple time scales whereas most previous studies have used short and fixed inter-stimulus intervals (ISI)^3,6–9,12,14^. Consequently, it is unknown whether there exists a neural correlate of statistical learning of transition patterns when ISI is long or variable. In the end, it is still not fully understood how transition patterns are learned and encoded in the auditory system^3,15^.

**Figure 1:**
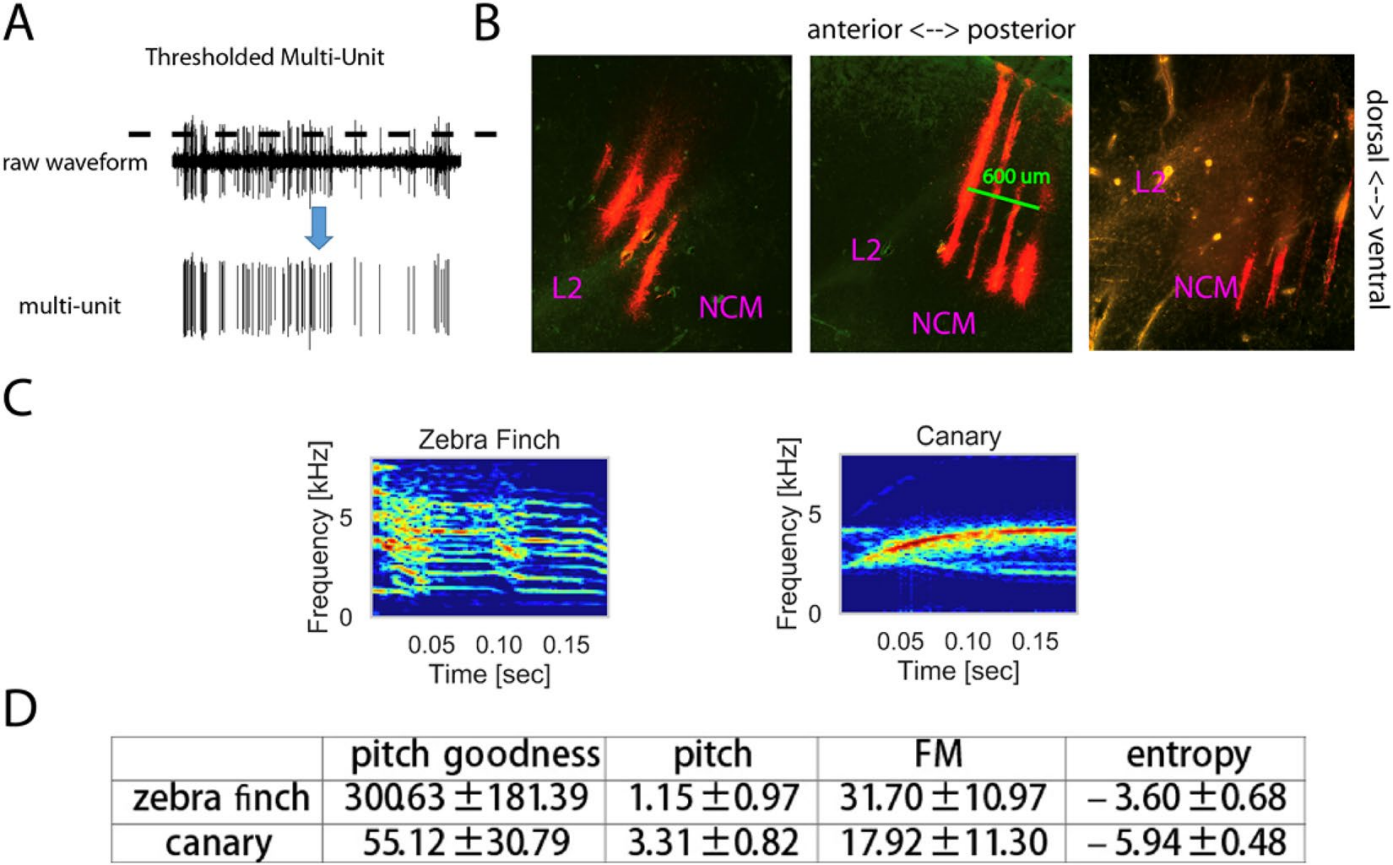
Thresholded multi-unit activity, histology, and syllable stimuli. (**A**) Illustration of how multi-unit activity (MUA) was obtained by thresholding the raw waveforms (3 standard deviations above the mean). (**B**) Histological verification of recording sites. Each image is a sagittal section from one bird (left is anterior while right is posterior; ~ 1 mm from the midline). In each image, the light green diagonal line indicates Field L2 and the 4 bright red lines show the electrode traces. In total, 186 and 161 responsive sites were recorded from NCM and Field L2, respectively. (**C**) Example spectrograms of one zebra finch syllable and one canary syllable. (**D**) Major acoustic differences between zebra finch and canary syllables used in the current experiment, measured using Sound Analysis Pro^41^. Pitch is measured in kHz; entropy does not have a unit; FM is the angular component of squared time and frequency components; pitch goodness has units comparable to Amplitude^41^.

**Figure 2:**
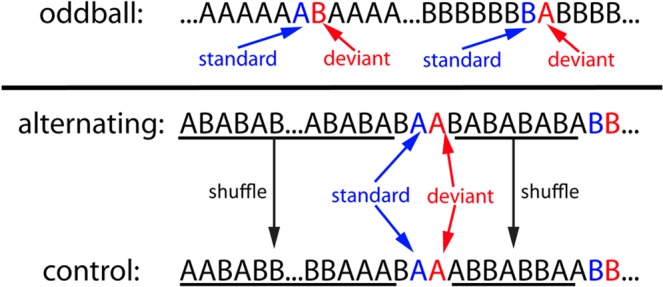
Classical oddball paradigm and alternating oddball paradigm. In the classical oddball paradigm, two stimuli were presented in two blocks with different probabilities. For notation purposes, when a stimulus is presented with low probability, it is called the deviant and the stimulus immediately preceding it is called the standard. Deviant and standard are color-coded with red and blue, respectively. The alternating oddball paradigm includes the alternating and control condition. In the alternating condition, two stimuli (**A,B**, one canary and one zebra finch syllable) were initially presented in alternation 25 times to familiarize the bird with the stimuli and the alternation pattern. Then, rare repetitions were presented after a variable 4–10 regular alternations. The deviant (2nd stimulus in the repetition), standard (1st stimulus in the repetition), and the stimulus immediately before them formed a “triplet.” In the control condition, the number of stimulus trials and the positions of the triplets were the same as those in the alternating condition, however, stimulus sequences between the triplets were shuffled in the control condition.

To study whether the auditory system can detect an oddball stimulus when its onset is not fully predictable, we conducted the classical oddball experiment at a jittered ISI. To study whether the auditory system is sensitive to violations of transition patterns at long and variable ISI, we used the alternating oddball paradigm at 3 different ISIs. We recorded extracellular activity in the male zebra finch auditory forebrain using an electrode array (4 shanks * 4 sites with 200 um spacing) (Fig. 1B). The zebra finch is one of the best-developed animal models to study these questions because they produce complex vocalizations for vocal communications, providing a repertoire of related, salient, but distinct experimental stimuli, and their auditory system has been well-studied^16,17^. Note that only male zebra finches were used in the current experiment and females may behave differently. The neural responses to zebra finch and canary song syllables (Fig. 1C) were measured with both thresholded multi-unit activity (MUA) and automatically sorted single-unit activity (SUA) (Fig. 1A)^18,19^. In the classical oddball paradigm, one stimulus is presented with low probability as deviant while the other stimulus is presented with high probability as standard (Fig. 2A). In the current experiment, the ISI was jittered between 0.8 and 1.2 s. Consequently, the interval between two consecutive stimuli changed from trial to trial and the onset of a stimulus was not fully predictable. The alternating oddball paradigm includes one alternating and one control condition. In the alternating condition, rare repetitions are presented after a sequence of alternating sounds; the 1st stimulus of the repetition is standard because it follows the alternating pattern whereas the 2nd stimulus of the repetition is deviant because it violates the alternating transition pattern of the preceding sequence. In the control condition, the sound sequence was shuffled, so that a repeated stimulus was not deviant^15^. In this case, stimuli were repeated at the same point in the overall sequence as for the alternating condition; the 1st and 2nd sound in the repetition were labeled as standard and deviant, respectively. The alternating and control condition were conducted using three different ISIs: fixed 1 s, fixed 3 s, and jittered (0.8 to 1.2 s). Based on previous results about repetition suppression and deviance detection^9,20,21^, we expect: 1) in the classical oddball condition at jittered ISI, a stimulus elicits larger neural responses as oddball than as standard; 2) in the control condition, a stimulus elicits smaller neural responses as deviant (2nd stimulus in the repetition) than as standard (1st stimulus in the repetition); 3) in the alternating condition, the neural responses to the deviant are larger than expected in NCM but not in Field L2 at all tested ISIs because previous studies have shown higher auditory areas are more sensitive to regularities in the sound sequence than primary auditory areas^8,22^.

Our MUA results showed that neurons were sensitive to violations of transition patterns at all three ISIs in the caudomedial nidopallium (NCM, secondary auditory area) but not in Field L2 (primary auditory area). These results demonstrate a neural correlate of statistical learning of transition patterns between sounds at multiple time scales and suggest that primary and higher auditory areas may play different roles in encoding transition patterns. The SUA results showed that neurons, specifically those with narrow spikes, were sensitive to violations of transition patterns at 1 s ISI in NCM but not in Field L2, suggesting that different types of neurons may play different roles in deviance detection. The MUA results also suggest that neural oscillation may be one mechanism of encoding transition pattern when ISI is short (1 s) and fixed, which is consistent with previous studies^5,23–25^. Together, our results suggest that different neural mechanisms may underlie the prediction of future events at different temporal scales. These results also provide new evidence for the predictive coding hypothesis^26,27^, which states that the nervous system constantly tries to predict future stimuli. Because prediction for future stimuli can help reduce the uncertainty of a sound (e.g., word) in a rapid series of sounds (e.g., speech) when individual sounds are noisy (e.g., in a noisy environment), our results may also provide insight into the neural mechanisms of rapid speech processing.

## Results

### Classical oddball effects exist in both Field L2 & NCM at jittered ISI

In the oddball condition, a rare deviant was presented after a repeating standard sound at a jittered inter-stimulus interval (ISI) (0.8 to 1.2 s; see Methods for details). The surprise index (SI) was used to quantify differences in neural responses to the deviant and standard (see Methods). A positive SI indicates that neural responses to the deviant are larger than to the standard, whereas a negative SI indicates that responses to the deviant are smaller than to the standard.

**Figure 3:**
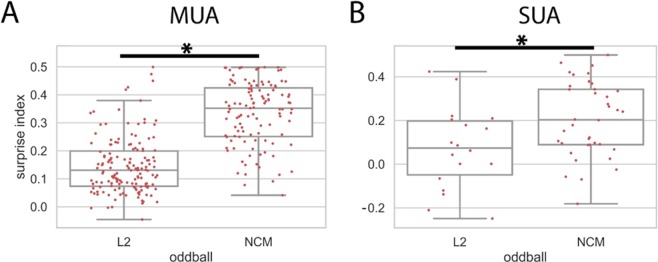
Surprise index (SI) in Field L2 and NCM at jittered inter-stimulus intervals (ISI). (**A**) Multi-unit activity (MUA). Each dot represents SI from one recording site. The SI was significantly larger than 0 in both L2 and NCM and was significantly larger in NCM than in L2. The box shows the quartiles of the dataset while the whiskers extend to the rest of the distribution, except for a few potential outliers. A horizontal line with a star indicates that a comparison is significant. (**B**) Single-unit activity (SUA). The SI in NCM was significantly larger than 0 and larger than that in L2. Note that SIs were compared across different units using the independent sample t-test because single-units cannot be held across different recording conditions (see Methods). Note that y-axis has different scales in MUA and SUA.

For multi-unit activity (MUA), the SI was significantly larger than 0 in both Field L2 and NCM (NCM: t = 27.834, p < 0.001, n = 115; Field L2: t = 17.102, p < 0.001, n = 150; one-sample t-test), suggesting that a stimulus elicits larger neural responses when it is the rare deviant than when it is the frequent standard (Fig. 3A). The SI was significantly larger in NCM than in Field L2 (t = 12.209, p < 0.001, n1 = 115, n2 = 150; independent sample t-test), suggesting that neural responses to a sound are more sensitive to the occurrence probability of a sound in NCM than in Field L2. This result is consistent with previous findings that the oddball effect is stronger in secondary auditory regions than in primary auditory regions^10,11,13^. The data show an additional phenomenon: the oddball effect exists even when ISI is variable and onset of a stimulus is not fully predictable (varies randomly between 0.8 and 1.2 seconds).

For single-unit activity (SUA), the SI in NCM was significantly larger than 0 (t = 7.455, p < 0.001, n = 38; one sample t-test) and larger than that in Field L2 (t = 2.659, p = 0.010, n1 = 38, n2 = 18, independent sample t-test) (Fig. 3B). However, the SI in Field L2 was not different from 0 (t = 1.626, p = 0.122, n = 18; one sample t-test) (potentially due to small sample size).

### Neural responses are not sensitive to transition patterns in Field L2 in the alternating oddball paradigm

**Figure 4:**
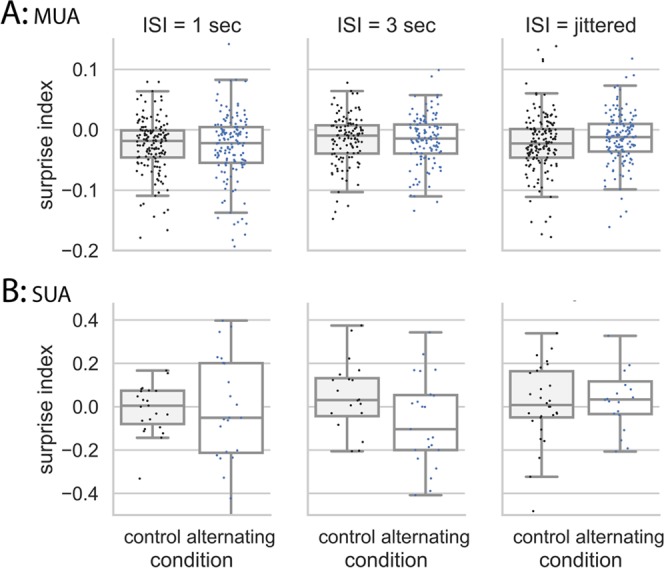
SI in the control and alternating conditions in Field L2 at 1 s, 3 s, and jittered ISI. (**A**) MUA. When ISI was fixed (1 s or 3 s) or jittered (0.8–1.2 s), SI in the alternating conditions were not different from the control condition, and SI was significantly smaller than 0. Note that SIs were compared within the same recording sites between the control and alternating condition using the Wilcoxon test and the scale in the y-axis is smaller here than in Fig. 5. (**B**) SUA showed the same results as MUA. Note that SIs were compared across different units using the Mann-Whitney U test because single-units cannot be held across different recording conditions. Note that some data points in the bottom left figure were cut off to have a proper scale across the three figures. In the statistical analyses, all data points were included.

MUA results in Field L2 showed that the SI in the control condition was significantly smaller than 0 at all three ISIs (W > 2410, p < 0.001 for all comparisons, n = 155, 132, and 161 for 1 s, 3 s, and jittered ISI, respectively; see Methods for use of Wilcoxon test). These results showed that neural responses to the 2nd stimulus in the repeated pair were smaller than to the 1st stimulus, regardless of the ISI tested (Fig. 4A). This suppression effect lasted at least 3 seconds and occurred even when ISI was jittered (0.8 to 1.2 s). Because the stimulus sequence was random and the 2nd stimulus in the repeated pair did not violate any regularities (neither expected nor unexpected), these results suggest that repetition suppresses neural responses to a stimulus in the absence of expectation and neural responses may habituate to a repeated stimulus. The SI in the alternating condition was not significantly different from that in the control condition at all tested ISIs (1 s ISI: W = 4290, p = 0.710, n = 133; 3 s ISI: W = 3241, p = 0.567, n = 117; jittered ISI: W = 5120, p = 0.0760, n = 156; Wilcoxon test). For the jittered ISI, the SI was slightly larger in the alternating condition than in the control condition but was not statistically significant.

Results from SUA were similar to those seen in MUA (Fig. 4B). The SI in the control condition was not different from 0 at all three ISIs (1 s ISI: W = 125, p = 0.692, n = 24; 3 s ISI: W = 130, p = 0.568, n = 24; jittered ISI: W = 193, p = 0.281, n = 31; Wilcoxon test). The SI in the alternating condition was not significantly different from that in the control condition at all tested ISIs (1 s ISI: U = 266.5, p = 0.328, n1 = 24, n2 = 24; 3 s ISI: U = 189, p = 0.117, n1 = 24, n2 = 20; jittered ISI: U = 325, p = 0.5, n1 = 21, n2 = 31; Mann–Whitney U test).

### Neural responses are sensitive to transition patterns at multiple temporal scales in NCM in the alternating oddball paradigm

**Figure 5:**
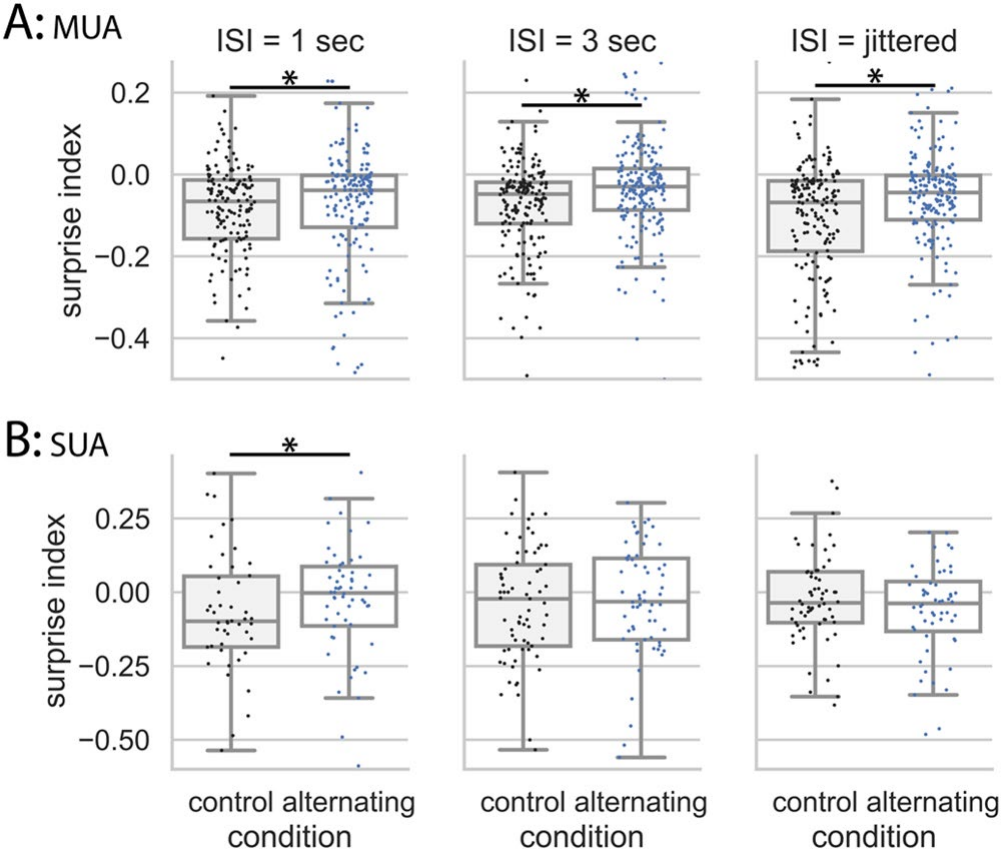
SI in the control and alternating conditions at 1 s, 3 s, and jittered ISI in NCM. (**A**) MUA. At all ISIs, the SI in the alternating condition was significantly larger than in the control condition. The SI in the control conditions was significantly smaller than 0. Note that SIs were compared within the same recording sites using the Wilcoxon test (with-group comparison). (**B**) SUA. SI in the alternating condition was significantly larger than in the control condition at 1 s ISI, but not at 3 s or jittered ISI. Note that SIs were compared across different units using the Mann-Whitney U test because single-units cannot be held across different recording conditions (between-group comparison).

The MUA in NCM showed different behavior from MUA in Field L2 (Figs. 4A, 5A). The SI in the alternating condition was significantly larger than in the control condition at 1 s (W = 3811, p = 0.0011, n = 148; Wilcoxon test), 3 s (W = 5510, p < 0.001, n = 181), and jittered ISI (W = 4836, p = 0.0045, n = 182), even though the SI in the alternating condition was still significantly smaller than 0 (1 s ISI: W = 3577, p < 0.001, n = 169; 3 s ISI: W = 4513, p < 0.001, n = 188; jittered ISI: W = 2607, p < 0.001, n = 175; Wilcoxon test) (Fig. 5A). These results showed that the responses to the deviant 2nd stimulus in the repeated pair were larger than usual, suggesting that these neurons detected the violation of the alternating pattern. This enhancement effect lasted at least 3 seconds and was also seen for jittered ISI (0.8 to 1.2 s). In the control condition, the SI was significantly smaller than 0 at 1 s (W = 1399, p < 0.001, n = 156; Wilcoxon test), 3 s (W = 2256, p < 0.001, n = 186), and jittered ISI (W = 1691, p < 0.001, n = 172). Also, the SI was significantly more negative in NCM than in Field L2 regardless of ISI (t = −10.157, p < 0.001, generalized linear mixed models, with area as fixed effects and ISI as random effects), suggesting that the repetition suppression is weaker in Field L2 than in NCM (Figs. 4A, 5A). Together, these results suggest that neurons in NCM are sensitive to transition patterns between sounds over multiple time scales and could detect deviants that violate transition patterns in the preceding stimulus stream.

In NCM, results from single-unit activity (SUA) were noisier than those from MUA (Fig. 5B). The SI was significantly larger in the alternating condition than in the control condition at 1 s ISI (U = 1263.5, p = 0.0275, n1 = 64, n2 = 50; Mann–Whitney U test), but not at 3 s or jittered ISI (3 s ISI: U = 2322.5, p = 0.358, n1 = 66, n2 = 73; jittered ISI: U = 1880.5, p = 0.153, n1 = 60, n2 = 70; Mann–Whitney U test). In the control condition, the SI was significantly smaller than 0 at 1 s ISI (W = 395, p = 0.0192, n = 50; Wilcoxon test) but not at 3 s or jittered ISI (3 s ISI: W = 1116.5, p = 0.198, n = 73; jittered ISI: W = 1065, p = 0.299, n = 70; Wilcoxon test). In the alternating condition, the SI was significantly smaller than 0 at jittered ISI (p = 0.0208, n = 60; Wilcoxon test) but not different from 0 at 1 s or 3 s ISI (1 s ISI: W = 1036, p = 0.981, n = 64; 3 s ISI: W = 992, p = 0.468, n = 66; Wilcoxon test).

**Figure 6:**
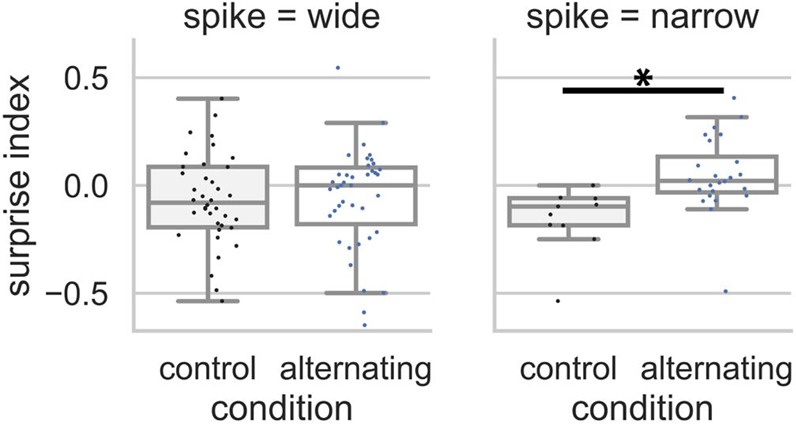
Surprise index from the narrow and wide spikes in NCM at 1 s ISI. For units with a wide spike waveform, SI in the control condition was not different from the alternating condition (U = 827.5, p = 0.255, n1 = 43, n2 = 42; Mann–Whitney U test). In contrast, for units with a narrow spike waveform, SI in the alternating condition was significantly larger than in the control condition (U = 49, p = 0.002, n1 = 24, n2 = 11; Mann–Whitney U test).

In NCM, after single units were classified into narrow and wide type based on their spike waveforms (see Methods for details), the SI was significantly larger in the alternating than in the control condition at 1 s ISI from the narrow spike units (putatively inhibitory) but not the wide spike units (putatively excitatory) (narrow: U = 49, p = 0.002, n1 = 24, n2 = 11; wide: U = 827.5, p = 0.255, n1 = 43, n2 = 42; Mann–Whitney U test) (Fig. 6). No difference in SI between units with narrow and wide spikes was found at 3 s or jittered ISI. In Field L2, no difference in SI was found between the alternating and control condition after single units were classified into wide and narrow types following the same method.

### A small subset of recording sites shows sign of neural oscillation

**Figure 7:**
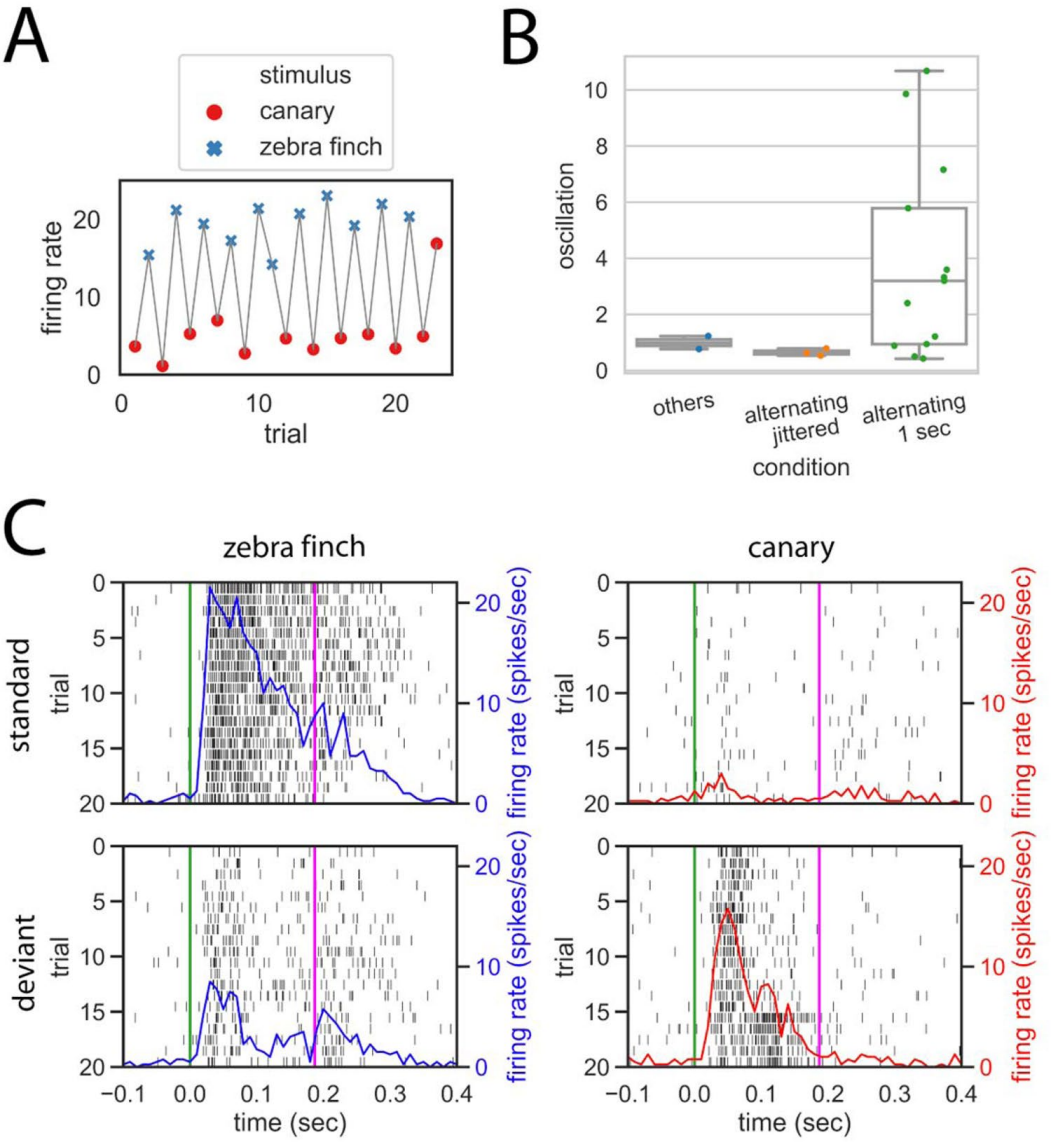
Schematic illustration of neural oscillation, oscillatory magnitude, and peri-stimulus time histogram (PSTH). (**A**) A recording site is oscillating if its responses are alternating between small and large as the two stimuli alternate and continue to alternate even when a stimulus repeats (see Methods for more detailed definition). Note that the neural response to the last canary syllable is more similar to the standard neural responses to the zebra finch syllable, as if the current stimulus was the zebra finch syllable (illustration). (**B**) Number of sites showing significant oscillation and corresponding oscillation magnitudes (see Methods for explanation). In the alternating condition at 1 s fixed ISI, 13 out of 341 (4%) recording sites showed oscillatory responses. In contrast, there were significantly less oscillatory sites in the alternating condition at jittered ISI or any other conditions combined. The oscillation magnitude was also significantly larger in the alternating 1 s condition than in any other conditions. Notice that the figure only shows sites that display significant oscillations. (**C**) Raster plot with overlying PSTH from one example recording site. Within each plot, the green and pink lines mark the stimulus onset and offset, respectively. The trial number (relative) goes from 1 to 20. The top left shows the raster plot and PSTH when a zebra finch syllable is a standard; the bottom left shows the raster plot and PSTH when the same zebra finch syllable is deviant. The figures on the right show the raster plot and PSTH when a canary syllable is standard (top) and deviant (bottom).

In our paradigm, the bird was passively exposed to the sound sequence without any external reinforcement, thus the transition patterns appear to be acquired via a statistical learning process, which may involve different neural mechanisms over the different time scales studied. Some of our observations suggest that the learned transition patterns may be encoded via neural oscillation when ISI is fixed at 1 s (Fig. 7). At some recording sites, the multi-unit activities (MUA) oscillated between small and large as the two stimuli (canary and zebra finch syllable) alternated (Fig. 7A). When one stimulus was repeated after frequent alternations, neural responses at those sites behaved as if the stimuli were still alternating. This continued alternation reponses can be a sign of neural oscillation, which may potentially encode transition patterns. In the alternating condition at 1 s ISI, 13 out of 341 recording sites (4%) showed oscillatory behavior (Fig. 7C) (see Method for detailed explanations). In contrast, we found significantly fewer oscillatory sites when ISI was jittered (p = 0.01, χ2 test) or 3 s (p < 0.01, χ2 test). In the control (random order) condition, no such sites were found (p < 0.01, χ2 test). The oscillation magnitude was also significantly larger in the alternating 1 s condition than in any other conditions (p < = 0.04, n1 = 13, n2 = 3; Mann–Whitney U test; see Method for the calculation of oscillation magnitude). Even though few recording sites showed sign of oscillation and further experiments may be needed to reach a final conclusion, our observations do suggest that neural responses at some recording sites may be entrained into an oscillation state by alternating stimulus and this oscillatory response may underlie the prediction of next stimulus at short, fixed ISI.

## Discussion

Our results based on MUA demonstrate a neural correlate of the statistical learning of transition patterns at multiple ISIs in the songbird auditory forebrain. Our SUA results further show that neurons with narrow spikes (putatively inhibitory) are sensitive to transition patterns at 1 s ISI. Furthermore, neural responses become more sensitive to the violation of transition patterns from primary auditory area Field L2 to secondary auditory area NCM. In thalamo-recipient Field L2, neural responses were sensitive to the probability of occurrence of a sound but not to the transition patterns between sounds. In contrast, neurons in NCM were sensitive to both.

Both L2 and NCM showed a classical oddball effect when ISI was variable (jittered). This result suggests that the auditory system can detect an oddball stimulus even when its onset is not fully predictable. This capability can help animals detect potential dangers (e.g., predators) in a complex environment in which the timing of a deviant sound is not always predictable. Past studies investigating deviance detection at the neural level have mostly used the oddball paradigm at a fixed ISI^10,11,28–30^. To our knowledge, ours is the first demonstration that an oddball effect is also elicited at a variable ISI. In addition, we found that the oddball effect was stronger in NCM than in L2, suggesting that the oddball effect in NCM does not fully originate in L2. One possible explanation for the oddball effect is that repetition suppresses the neural responses to the standard whereas deviance enhances the neural responses to the oddball. If this is the case, the magnitude difference in the effect between NCM and L2 may stem from how repetition and deviance differently influence the neural responses in NCM and L2.

MUA results show that simple repetition suppresses neural responses at multiple time scales in both L2 and NCM (SI < 0; Figs. 4A & 5A), but the repetition suppression is stronger in NCM than in L2. This result suggests that further neural processing may take place in NCM or in the projection pathway from L2 to NCM. The repetition suppression in L2 may be similar to repetition suppression observed early in the auditory system in mammals and share similar neural mechanisms^31,32^. In contrast, the stronger suppression in NCM may in addition reflect the memory trace of a sound, especially because other studies have shown that stimulus-specific adapted responses in NCM can persist over a long-time scale (hours to days)^20,21,33^. In the classical oddball paradigm, the stronger suppression in NCM may result in smaller responses to the standard than in L2, and at least partially explain why the oddball effect is greater in NCM.

The sensitivity to the violation of transition patterns demonstrates a neural correlate of statistical learning because detecting a repeated stimulus as a deviant requires the auditory system to learn the alternating transition patterns in the preceding stimulus sequence that the bird heard. In the alternating condition, the repeated stimulus deviates from the ongoing alternating pattern in the preceding sound sequence and thus is unexpected. In NCM, the neural responses to the 2nd stimulus in the repetition were larger in the alternating condition than in the control condition at 1 s ISI and provide evidence of deviance detection. For the MUA, enhanced responses were also seen for 3 s and jittered ISI, suggesting that the expectation of alternation lasted at least that long, and was also present for jittered ISI. These results show that the deviant sound in a complex sequence is detected even when the ISI is long or variable (jittered) and add to published findings that neurons in the auditory system are sensitive to transition patterns at short and fixed ISI^8,22^. The classification of SUA based on spike waveforms showed that only narrow (putatively inhibitory) neurons in NCM were sensitive to the violation of transition patterns at 1 s ISI whereas the wide (putatively excitatory) neurons were not. Because narrow (inhibitory) neurons in NCM mostly receive local connections, their activities may be more affected by neural activities from nearby neurons in NCM than neural activities in Field L2^34^. In contrast, the wide (excitatory) neurons may receive more connections from Field L2 and their activities may be heavily influenced by neural activities in L2. These connection differences between narrow and wide neurons in NCM may be the cause for the differences in sensitivity to transition violation. This result suggests that inhibitory and excitatory neurons play different roles in the statistical learning of transition patterns at least at 1 s ISI. In Field L2, no significant differences between alternating and control condition were seen. Together, these results suggest that sensitivity to the violation of transition patterns may emerge along the projection from primary auditory area (Field L2) to higher auditory area (NCM), consistent with previous reports^2,8,22,35,36^.

Our results also suggest that at least some neurons may encode learned transition patterns via neural oscillation when ISI is fixed at 1 s (Fig. 7). If other neurons in NCM receive both oscillatory prediction and stimulus-locked inputs (e.g. from L2), they could detect a deviant sound by comparing the two different types of responses. Our observation of oscillatory activity is consistent with previous reports that statistical learning of sequence can affect neural oscillation^5,23^ but more directly shows how neural oscillation may affect neural responses to a stimulus. However, we observed very few oscillatory sites and therefore more experiments are needed to verify this hypothesis. Also, NCM neurons were sensitive to violations of alternation patterns with 3 s or jittered ISI when neural oscillation was not observed, which suggests that oscillation is at most one of the mechanisms that encode transition patterns. When ISI is long or variable, other processes like state-dependent computation may contribute to encoding the transition patterns^2,8,14,22,35–37^.

In our experiments, all instances of a stimulus were identical. However, it would be valuable to investigate whether NCM neurons are sensitive to violations of transition patterns when different instances of a stimulus contain variations. For example, natural vocalizations of a sound are often variable even when produced by the same individual. Testing whether NCM neurons can acquire transition patterns between naturally-varying instances of sounds has the potential to provide a more ethological model for statistical learning of transition patterns during speech/language acquisition. Another limitation is that only male zebra finches were used in the current experiment and it remains to be tested whether female zebra finches show similar results.

In summary, our results show that neural responses in NCM are sensitive to the violation of transition patterns between sounds and demonstrate a neural correlate of statistical learning at multiple temporal scales. The alternating oddball paradigm is one example of using a simple artificial grammar to study auditory pattern processing^3,38^. Because learning transition patterns between sounds can facilitate rapid speech processing in noisy environments^4,39^, further work studying the neural mechanisms underlying these phenomena may provide insight into the treatment of certain auditory processing disorders.

## Methods

Some parts of the methods section are reproduced from an earlier publication^9^ by Dong & Vicario.

### Subjects

This study used 16 adult (>120 days) male zebra finches. All birds were obtained from a local vendor and housed in a general aviary with other zebra finches at Rutgers University under a 12 h: 12 h light/dark cycle and provided with water and food *ad libitum*. All experiments and methods were performed in accordance with relevant guidelines and regulations. All experimental procedures were approved by the Institutional Animal Care and Use Committee of Rutgers University.

### Surgery

Birds were prepared for electrophysiological recording under isoflurane anesthesia (1–2% in oxygen). The anesthetized bird was placed in a stereotaxic device, feathers on the scalp were removed and 0.04 cc Marcaine (0.25%) was injected under the scalp for local anesthesia. Then, a midline horizontal incision was made and enlarged to expose the skull. The outer layer of the skull was removed over the region of interest around the bifurcation of the mid-sagittal sinus. Dental cement was then used to form a small round chamber over the opening, and a metal pin was attached to the skull to keep the bird’s head fixed during subsequent awake electrophysiological recording. The bird received an injection of 0.04 cc Metacam (5 mg/mL) for post-operative analgesia and was closely monitored for recovery.

### Electrophysiological recording

After two days of recovery, birds were recorded in a walk-in sound attenuation chamber (Industrial Acoustics Company, Bronx, NY). The bird was restrained in a custom tube and fixed to the stereotaxic frame by clamping the previously implanted pin. A small craniotomy exposed the dura over the recording area. Two silicon probes (NeuroNexus, Ann Arbor, MI), one for each hemisphere, were lowered into the auditory forebrain (1.2 mm below the brain surface, 1 mm lateral from midline, 1.5 mm rostral to Y-point where cerebellum and both hemispheres meet). Each probe had 4 shanks and 16 recording sites (0.4–1 MΩ impedance at 1 kHz) in a 4-by-4 grid layout (Fig. 1B). Neighboring shanks are 200 um apart from each other and neighboring electrodes are 200 um apart within each shank. The probes were implanted in a para-sagittal plane such that the 4-by-4 grid layout is perpendicular to left-right (lateral) axes. Each probe was used for the right hemisphere for half of the birds and for the left hemisphere for the other half. Prior to insertion, the probes were dipped into a DiI solution (10% in ethanol; Sigma Aldrich, St. Louis, MO) and allowed to dry to label probe insertion tracks for later histological analyses. Figure 1B shows the location of recording probes along with the two main structures of the auditory forebrain: field L2 and Caudomedial Nidopallium (NCM). Field L2 is analogous to the primary auditory cortex in mammals, and NCM is similar to the superficial layer of the primary auditory cortex or the secondary auditory cortex in mammals^40^. Most sites (>95%) were assigned to NCM and Field L2 based on their relative location from Field L2 and sites from other recording areas were excluded in the analysis. In few cases (<5%) where anatomical data are unclear/missing from sectioning, sites were assigned to NCM and Field L2 based on the location of nearby sites and response characteristics during the baseline period if applicable.

All stimuli were equated for RMS amplitude and played back at a peak amplitude of 65 dB SPL (“A” scale) from a speaker placed 30 cm in front of the bird aligned with the midline. Once most electrodes showed spontaneous neural activities characteristic of the target area, playback of experimental stimuli began. Field L2 has spontaneous spikes with large amplitudes whereas NCM has spikes of smaller amplitudes. Two signal processors (Power 1401, CED, Cambridge, England) were used for stimulus presentation and neural recording. Neural activity was amplified (x 10,000), filtered (0.5–5 kHz bandpass), digitized (25 kHZ), and stored for further analysis.

Multi-unit activity (MUA) was obtained by thresholding the raw waveforms (3 standard deviations above the mean) for each recording site (Fig. 1A). Single unit activity (SUA) was discriminated by feeding the raw waveforms into the automatic spike sorting algorithm waveclus^18,19^. Sorted single units were included in the analysis only if the percentage of inter-spike intervals less than 2 ms (contamination rate) was less than 2%. For each electrode/unit, the neural response to each stimulus trial was computed by subtracting the average firing rate during the baseline period (1/4 of the inter-stimulus interval before the stimulus) from the firing rate during the stimulus period (plus 10% of stimulus duration).

### Auditory stimuli

The stimuli were syllables from zebra finch songs (recorded in our laboratory) and canary songs (recorded from our laboratory and sampled from on-line resources). We used these syllables to investigate how auditory system processes complex sounds instead of simple pure tones. Based on the measurements from Sound Analysis Pro^41^, zebra finch and canary song syllables had different acoustic features and potentially belonged to different acoustic categories. Figure 1C shows examples of zebra finch and canary syllables and their major acoustic differences (e.g., frequency modulation, pitch, entropy). Within each condition (see Fig. 2), a zebra finch syllable and a canary syllable of the same duration (range 140–190 ms) were used. With 3 different conditions and 3 different inter-stimulus intervals for 2 conditions, each bird was exposed to at least 7 different pairs of zebra finch canary syllables (sometimes extra pair of stimuli was played when there were technical difficulties). The particular stimuli used for different conditions were counterbalanced across birds so that different stimuli occur in different conditions with equal probabilities.

### Alternating oddball paradigm

Stimuli were presented in both classical oddball paradigm and alternating oddball paradigm (Fig. 2). In the classical oddball condition, two stimuli (A & B) were presented in two blocks. In the 1 st block, stimulus A was presented after a variable 4 to 10 repetitions of stimulus B. In the 2nd block, the roles of the two stimuli were reversed. For notation purposes, the rare stimulus was called the deviant and the stimulus immediately preceding it was called the standard. The alternating oddball paradigm included 2 conditions: the alternating and control condition. In the alternating condition, the two stimuli were first presented in an alternating order for 25 times (…ABABAB…), then rare repetitions of one of the two stimuli (AA or BB) were presented after a variable 4 to 10 common alternations (…ABABABA**BAA**BAB…). In total, there were 20 AA repetitions and 20 BB repetitions. For the repeated pairs, the 2nd stimulus was called the deviant because it violated the alternating regularities from the preceding sequence, while the 1st stimulus was called the standard. In the control condition, the stimulus sequence was generated from the alternating condition: the deviant, standard, and the stimulus immediately before it was kept at the same position as a triplet whereas the stimulus sequence between the triplets was shuffled. Consequently, the 2nd stimulus in the repetitions was deviant in the alternating condition but not in the control condition (still called deviant for notation purposes).

Alternating and control conditions were conducted at 3 different inter-stimulus intervals (ISI): 1s fixed, 3 s fixed, 1 s jittered (randomly drawn between 0.8 and 1.2 s after each trial). 3 s fixed ISI tests whether neurons are sensitive to transition patterns when ISI is long. Jittered 1 s ISI tests whether neurons are sensitive to transition patterns when ISI is variable. The classical oddball condition was conducted at 1 s jittered ISI. Assume both x_1_ & x_2_ follow a uniform distribution from 0.8 to 1.2 s. The jittered ISI was randomly drawn from the average of two independent variables, each following an independent uniform distribution from 0.8 to 1.2 s

### Criterion for Responsive Sites/Units and Classification of Single units

Recording sites (multi-unit) and units (single-unit) were included for data analysis if they responded to at least one of the zebra finch or canary stimulus. Any given recording site or single-unit was considered to be responsive to a stimulus if these conditions were met:

1. The firing rate during the stimulus period was significantly different from that during the baseline period based on the paired Wilcoxon test (*p* < 0.05).
2. The average neural response to the stimulus was above baseline (firing rate > 3 spikes/s). Consequently, units and sites that showed suppressed responses during the stimulus presentation were excluded from the analyses.

Recent studies have suggested there may be two different types of neurons in the songbird auditory forebrain^22,42–45^. One type has narrower spike waveforms and is putatively inhibitory. The other type has wider spike waveforms and is putatively excitatory. Single-units from all recording sites and experimental conditions were pooled together and classified into narrow and wide types using the following procedure based on a previously established method^46,47^:

1. Calculate the average spike waveform for each unit and normalize the waveform by dividing it by its maximum.
2. Perform principal component analysis (PCA) for all the normalized spike waveforms and keep the first 4 components (explained more than 95% of all variances).
3. Use the K-means clustering algorithm to classify the waveforms into two classes with the 4 components as features.
4. The units with the wider average waveform are called wide and the rest are called narrow.

**Figure 8:**
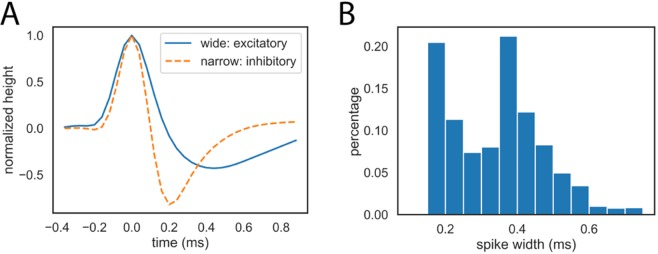
Spike waveform and width of wide- and narrow-spiking units. (**A**) Average spike waveform of the wide- and narrow-spiking units. The height of spikes is normalized to 1 during classification. (**B**) Distribution of the spike widths in milliseconds. There are two distinct peaks. Note that the accuracy of the spike width is limited by our sampling rate during recording time (25 kHZ).

Similar to previous reports^44^, our wide-spiking (WS) and narrow-spiking (NS) neurons differed in spike width (NS: 0219 ± 0.041 ms; WS: 0.461 ± 0.152 ms; Mann-Whitney U-test, P < 0.001; see Fig. 8).

### Quantify Neural Response Differences Elicited by the Deviant and the Standard

The surprise index (SI) quantifies differences in neural response to the deviant and standard^10^.

1 $${\rm{SI}}=\frac{{R}_{d}-{R}_{s}}{{R}_{d}+{R}_{s}}$$

In the alternating oddball experiment, *R*_*d*_ and *R*_*s*_ represent the average neural responses to two stimuli when they were deviant and standard. If the site/unit responded only to one of the stimuli, SI was calculated using the neural responses from the stimulus that elicits significant responses.

### Quantify the magnitude of neural oscillation

The magnitude of neural oscillation is estimated by how strong the neural responses continue the alternating pattern when the stimuli no longer alternate (e.g., repetition after many alternations). For each recording site in each condition, we calculated the responses to each stimulus as firing rate during stimulus presentation. For each stimulus, we measured its baseline responses when it is standard while excluding the 3 trials immediately after the deviants to remove potential post-effects from the deviant. If the baseline neural responses to the two stimuli (*S*_*x*_ and *S*_*y*_) were significantly different and *S*_*x*_ < *S*_*y*_, the neural responses to stimulus x and y will alternate between low and high as the two stimuli alternate in the sequence …xyxyxyxy… (see Fig. 7A). If neural oscillation (alternating responses entrained by the two alternating stimuli) affects neural responses strongly, then, when stimulus x repeats (e.g., …xyxyxyx**x**…), the neural responses to the 2^nd^ x in the repetition (*D*_*x*_) will be close to the neural responses to y when it is standard (*S*_*y*_) and *D*_*x*_ > *S*_*x*_ (as if the two stimuli were still alternating). In this case, (*D*_*x*_ − ${\bar{S}}_{x}$)/std(*S*_*x*_) measures a normalized oscillation magnitude. In other words, neural oscillation will drive the neural responses to the 2^nd^ stimulus in the rare repetition after frequent alternations towards the neural responses to the other stimulus when it is standard. Similarly, if *S*_*x*_ > *S*_*y*_ and neural oscillation strongly affects neural responses, the neural responses to the 2^nd^ x in the repetition (*D*_*x*_) will be smaller than *S*_*x*_. To have a positive neural oscillation magnitude in both cases, we quantify the oscillation magnitude using:

2 $${oscillatio}{{n}}_{{x}}=\{\begin{array}{cc}({D}_{x}-{\bar{S}}_{x})\div{\rm{std}}({{S}}_{{x}}) & if\,{\bar{S}}_{x} < {\bar{S}}_{y}\\ -({D}_{x}-{\bar{S}}_{x})\div{\rm{std}}({{S}}_{{x}}) & if\,{\bar{S}}_{x} < {\bar{S}}_{y}\end{array}$$

where, ${\bar{S}}_{x}$ and ${\bar{S}}_{y}$ are the average baseline neural responses when stimulus *X* and *Y* are standard; *D*_*x*_ is the response to *X* as deviant when it is in the repetition; std(*S*_*x*_) is the standard deviation of neural responses to stimulus *X* during baseline.

If a stimulus elicited small neural responses during baseline, its oscillation magnitude is positive when it elicited larger responses when it repeats as a deviant than during baseline (Fig. 7). If a stimulus elicited large neural responses during baseline, its oscillation magnitude is positive when it elicited smaller responses when it repeats as a deviant than during baseline. A recording site is oscillatory if the oscillation magnitudes of both stimuli are significantly larger than 0, because it indicates that the neural responses to the 2nd stimulus in the repetition are oscillating as if the stimuli were alternating. Because repetition suppression will increase the oscillation magnitude when a stimulus elicits strong responses and to remove potential effects from repetition suppression, the calculation of the average oscillation magnitude of a site only includes the stimulus that usually elicits small neural responses. In other words, oscillation magnitude is calculated based on the stimulus that elicits smaller neural responses as standard but elicits much larger neural responses as deviant.

### Histology

At the end of each recording experiment, the bird was sacrificed with an overdose of pentobarbital (0.15 ml of 39 mg/ml; Vortech Pharmaceutical, Dearborn, MI), and perfused with 0.9% saline and 3.3% paraformaldehyde. After several days of fixation, the brain was cut into 75 um sagittal sections using a Vibratome and visualized with an epifluorescence microscopy. Figure 1B shows the location of recording probes along with the two main structures of the auditory forebrain: field L2 and Caudomedial Nidopallium (NCM).

### Statistical Analyses

In the analysis using surprise index (SI), each sample is one site/unit and different statistical tests were used for comparisons based on MUA and SUA data. For MUA, we performed within-subject comparisons because comparisons are mostly within the same group of electrodes; for SUA, we performed between-subject comparisons because spike sorting was done separately for different experimental conditions and units from different conditions cannot be guaranteed to be the same. For within-subject comparisons, we used the paired sample *t*-test; for between-subject comparisons, we used the independent sample *t*-test; for comparisons with hypothesized population means, we used the one sample *t*-test. When the normality assumption was not met (Shapiro–Wilk test), corresponding non-parametric statistical tests (Wilcoxon test and Mann Whitney *U*-test) were used. The significance level was set at 0.05 (Bonferroni adjusted *p*-values were reported in the context oddball analysis using SI as 7 comparisons were conducted). Because we did not find any significant differences between the left and right hemisphere (t = 0.044, p = 0.965; generalized linear mixed model, with hemisphere as fixed effects and other factors as random effects), results were pooled together in all analyses. All analyses were conducted using customized scripts in Spike2, Matlab, R, and Python.

## Data Availability

The datasets generated during and/or analyzed during the current study are available from the corresponding author on reasonable request.
